# Systematic Analysis of the Oncogenic Role of WDR62 in Human Tumors

**DOI:** 10.1155/2021/9940274

**Published:** 2021-07-01

**Authors:** Yulan Bu, Lihua Zhang, Xiaolin Ma, Rui Wang, Xuecheng Zhang, Jiaqiu Li

**Affiliations:** ^1^Jinming Yu Academician Workstation of Oncology, Clinical Research Center, Affiliated Hospital of Weifang Medical University, Weifang 261031, China; ^2^Department of Radiotherapy, Affiliated Hospital of Weifang Medical University, Weifang 261031, China; ^3^Department of Oncology, Affiliated Hospital of Weifang Medical University, Weifang 261031, China

## Abstract

**Background:**

Emerging studies support the oncogenic role of WD repeat domain 62 (WDR62) in few tumors, while no pan-cancer analysis is available. In this study, we analyzed systematically the oncogenic role of WDR62 across a series of human tumors based on bioinformatic data mining.

**Methods:**

The expression level of WDR62 was analyzed via GEPIA2, TIMER, UALCAN, and StarBase databases. The prognostic role was analyzed via GEPIA2, TIMER, UALCAN, StarBase, TISIDB, TCGA portal, Kaplan-Meier Plotter, and PrognoScan databases. Then, we explored the causes for WDR62 abnormal expression via TCGA portal and UALCAN databases. Subsequently, the STRING and GeneMANIA databases were used to find the interactive networks for WDR62. Furthermore, we analyzed the correlation between WDR62 expression and immune features via TIMER and TISIDB databases.

**Results:**

We found that WDR62 was significantly upregulated in most of the tumors and correlated with poor prognosis mainly in 6 candidate tumors—BLCA, BRCA, KIRC, KIRP, LIHC, and LUAD. Abnormal WDR62 expression may be probably attributed to TP53 mutation and promoter DNA methylation. Relative network analysis demonstrated that WDR62 was mainly involved in MAPK and toll-like receptor signaling pathway. WDR62 expression was associated with various immune cell infiltrations, especially cancer-associated fibroblasts (CAF) and T cell regulatory (Treg) cells, and was markedly correlated with poor prognosis. Moreover, WDR62 expression was closely associated with the expression of some immunomodulators such as PD-L1 and has a significant prognostic value.

**Conclusions:**

Our study revealed that WDR62 could serve as a diagnostic and prognostic biomarker for several cancers. Importantly, WDR62 was closely associated with various immune cell infiltration, and to a certain extent, it can predict the effect of immunotherapy in particular PD1/PD-L1 inhibitors. Our pan-cancer study provided useful information on the oncogenic role of WDR62, contributing to further exploring the underlying mechanisms.

## 1. Introduction

According to the latest statistics report released by the International Agency for Research on Cancer (IARC), an estimate is approximately 19.3 million new cancer cases and 10.0 million cancer deaths in 2020 worldwide [1]. Among them, the incidence rate of breast cancer has surpassed that of lung cancer as the most diagnosed tumor for the first time. Despite great progress in cancer diagnosis and treatment, it is still not satisfactory in consideration of its leading threat to human life. Therefore, efforts to explore novel biomarkers for the diagnosis of cancers and the prediction of cancer therapeutic effect are critical for cancer control.

With the development of high-throughput sequencing technology, a lot of databases especially those based on TCGA (The Cancer Genome Atlas) and GEO (Gene Expression Omnibus) datasets have emerged [2, 3]. Consequently, it is convenient for us to conduct a pan-cancer analysis of genes of interest, promoting the discovery of new biomarkers. Targeting immune checkpoint, in particular PD1/PD-L1 inhibitors, has shown clinical activity in a wide spectrum of tumors such as lung cancer [4]. However, as an evolving treatment, PD1/PD-L1 inhibitors only benefited a limited number of patients, demonstrating the complexity of tumorigenesis. It is essential for the establishment of new biomarkers to identify patients who are suitable for immunotherapy. Meanwhile, some biomarkers may predict clinical response and account for the underlying resistance mechanism, providing guidance for immunotherapy in the clinic [5]. WDR62, a novel biomarker, is known to be responsible for the carcinogenesis of few tumors such as ovarian cancer and gastric cancer [6, 7]. These findings indicated that WDR62 overexpression was correlated with centrosome amplification and was confirmed as a novel prognostic biomarker. However, the oncogenic role of WDR62 in other cancers remains unknown. This study is aimed at analyzing systematically the oncogenic role of WDR62 across a series of human tumors based on bioinformatic data mining. Furthermore, the association between WDR62 expression and immune features was investigated in our study for the first time.

## 2. Materials and Methods

### 2.1. GEPIA2 Database Analysis

We used the “Box Plot” module of the GEPIA2 database [8] to search for the expression level of WDR62 in all cancer datasets. The ILog_2_FCI cutoff is 1. *P* value cutoff is 0.05. Jitter size is 0.4. Matched normal data contains TCGA normal and GTEx data. Besides, we obtained the WDR62 expression data in different stages via the “Stage Plot” module. We used the “Survival Map” module to acquire the overall survival (OS) and disease-free survival (DFS) map data of WDR62 across all tumors. The group cutoff is median by cutoff high (50%) and cutoff low (50%). Significance level is 0.05. Then, the “Survival Analysis” module was used to analyze the detailed OS and DFS data of single cancer. We obtained the hazard ratio (HR) and *P* value by the log-rank test. We used the “Correlation Analysis” module to analyze the correlation between WDR62 expression and CD274 (also known as PD-L1) expression in tumor tissues by Spearman correlation coefficient. *P* value cutoff is 0.05.

### 2.2. TIMER Database Analysis

We used the “Diff Exp” module of the TIMER database [9, 10] to dissect the expression level of WDR62 in different tumors. Statistical significance of different expression was evaluated using the Wilcoxon test. *P* value cutoff is 0.05. The “Survival” module allows us to compare the overall survival between high and low WDR62 expression level groups, under the setting of cutoff high (50%) and cutoff low (50%). The “Correlation” module was used to analyze the correlation between WDR62 expression and CD274 expression in tumor tissues by Spearman correlation coefficient. *P* value cutoff is 0.05. And we used the “Gene” module to analyze the association between WDR62 expression and immune cell infiltration. Immune cells contain B cell, CD4+ T cell, CD8+ T cell, macrophage, neutrophil, and dendritic cell. *P* value cutoff is 0.05. Subsequently, we continued to analyze the correlation between WDR62 expression and cancer-associated fibroblasts (CAF) and T cell regulatory (Treg) cells in the “Gene” module of TIMER2 (Tumor Immune Evaluation Resource, version 2). And we explored the overall survival in a multivariable Cox hazard model. Covariates are WDR62 expression and CAF or Treg infiltration levels. Increased risk was set as *P* value < 0.05 and *Z* score > 0. The data was visualized as a heat map and a scatter plot. Moreover, we used the “Gene-Corr” module to draw the heat map of relative genes in different tumors. *P* value cutoff is 0.05.

### 2.3. UALCAN Database Analysis

The mRNA expression of WDR62 in tumor and normal tissues was determined within the UALCAN database [11]. And we compared WDR62 expression in breast cancer among different stages, races, ages, subclasses, histologic subtypes, nodal metastasis status, and TP53 mutation status. Moreover, the overall survival data and promoter methylation status of WDR62 in candidate tumors were analyzed. *P* value cutoff is 0.05.

### 2.4. StarBase Database Analysis

The expression of WDR62 in tumor and normal tissues was determined within the StarBase V3.0 database [12]. And the overall survival data of WDR62 in different cancers was analyzed. Moreover, the correlation between WDR62 expression and CD274 expression in tumor tissues was explored. *P* value cutoff is 0.05.

### 2.5. TISIDB Database Analysis

The TISIDB database was used to analyze the association between WDR62 expression and overall survival and stage data across the candidate tumors [13]. Furthermore, we compared the correlation between WDR62 expression and immunoinhibitors. And the correlation between WDR62 expression and CD274 or CTLA4 expression in tumor tissues was analyzed by Spearman correlation coefficient. *P* < 0.05 was considered statistically significant. Finally, we compared the WDR62 expression difference between responders and nonresponders receiving PD1/PD-L1 inhibitors. *P* < 0.05 was considered statistically significant.

### 2.6. TCGA Portal Database Analysis

The correlation between WDR62 expression and prognostic significance was analyzed by specialized prognostic database—TCGA portal [14]. The overall survival (OS) data was obtained based on TCGA datasets. *P* value < 0.05 was considered statistically significant.

### 2.7. Kaplan-Meier Plotter Database Analysis

Kaplan-Meier Plotter is able to assess the survival data based on GEO, EGA, and TCGA datasets, promoting the discovery and validation of survival biomarkers [15]. The prognostic value of WDR62 expression including the overall survival (OS), disease-free survival (DFS), first progression survival (FP), and relapse survival (PPS) was evaluated. Furthermore, the WDR62 prognostic value along with immune cell infiltration including CD4+ T cell, CD8+ T cell, macrophage, natural killer (NK) T cells, and regulatory T cells (Treg) was compared. *P* value < 0.05 was considered statistically significant.

### 2.8. PrognoScan Database Analysis

The correlation between WDR62 expression and prognostic significance was analyzed by specialized prognostic database—PrognoScan [16]. The overall survival (OS), disease-specific survival (DSS), and disease-free survival (DFS) data were obtained based on the GEO datasets. *P* value < 0.05 was considered statistically significant.

### 2.9. STRING Analysis

The protein-protein interaction (PPI) networks of WDR62 were analyzed by online tool STRING [17]. We set the parameters as follows: network type (full STRING network), meaning of network edges (evidence), active interaction sources (experiments), minimum required interaction score (low confidence (0.150)), and max number of interactors to show (no more than 50 interactors).

### 2.10. GeneMANIA Database Analysis

The GeneMANIA database was used for exploring the interaction network for WDR62 [18, 19]. The bioinformatic methods involved gene physical interaction, coexpression, prediction, colocation, pathway, genetic interaction, and shared protein domains. We visualized the top 20 gene networks through the GeneMANIA.

### 2.11. DAVID Analysis

The interactive genes of WDR62, conducted by STRING and GeneMANIA databases, were all input into Database for Annotation, Visualization, and Integrated Discovery (DAVID) online tool for Gene Ontology (GO) and related pathway analysis [20]. GO analysis contains biological process (BP), molecular function (MF), and cellular component (CC). Related pathways contain Kyoto Encyclopedia of Genes and Genomes (KEGG) pathways and Reactome pathways. *P* value < 0.05 was considered statistically significant.

### 2.12. Statistics

Student's *t*-test was performed for statistical significance analysis. *P* value < 0.05 was considered as statistically significant; ^∗^*P* < 0.05, ^∗∗^*P* < 0.01, ^∗∗∗^*P* < 0.001, and ^∗∗∗∗^*P* < 0.0001.

## 3. Results

### 3.1. Gene Expression Analysis Data

**Figure 1:**
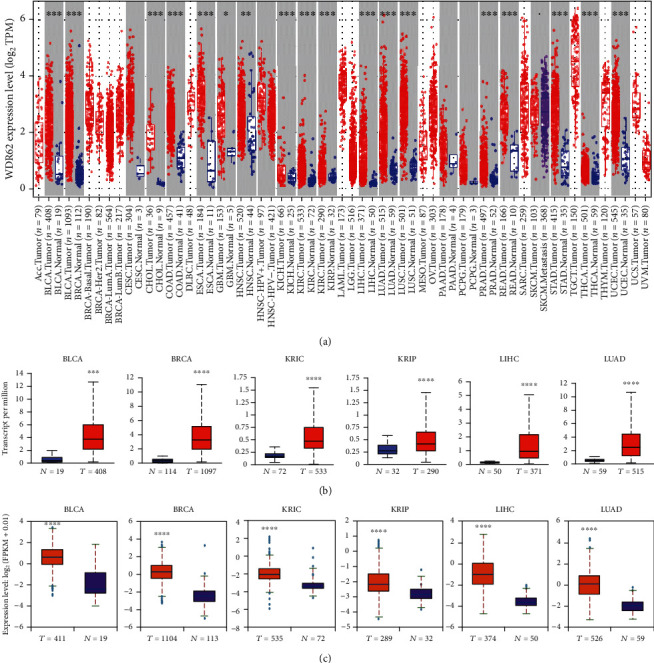
Expression level of WDR62 in different tumors. (a) The expression level of WDR62 in different tumors or specific tumor subtypes was analyzed through the TIMER database. (b) The expression level of WDR62 between normal tissue and primary tissue of BLCA, BRCA, KIRC, KIRP, LIHC, and LUAD in the UALCAN database. (c) The expression level of WDR62 between normal tissue and primary tissue of BLCA, BRCA, KIRC, KIRP, LIHC, and LUAD in the StarBase database.

**Figure 2:**
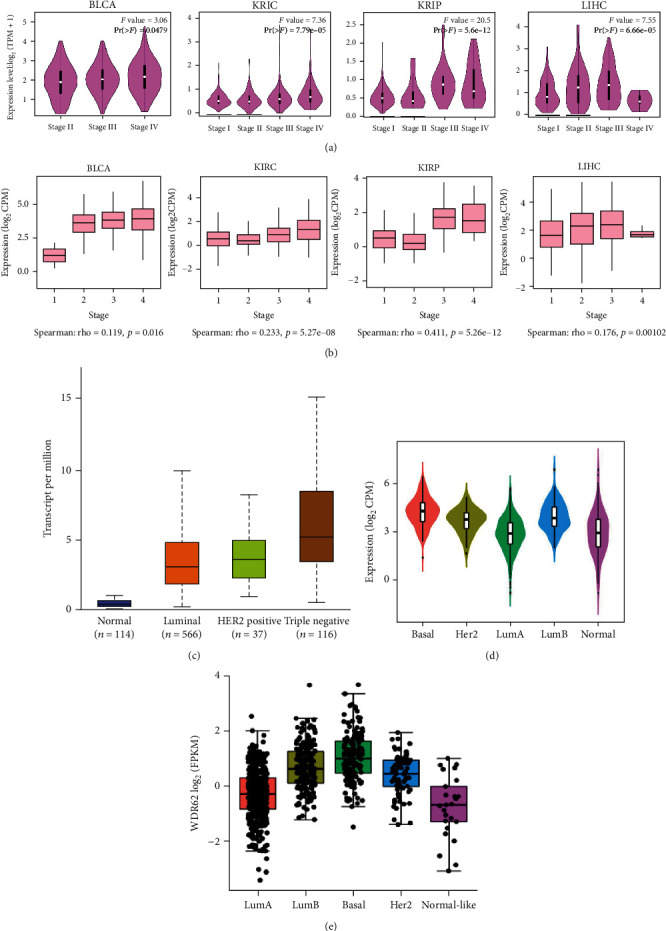
Expression level of WDR62 in different pathological stages and subtypes. (a, b) Based on TCGA data in the GEPIA2 and TISIDB database, the expression levels of WDR62 were analyzed in the main pathological stages of BLCA, KIRC, KIRP, and LIHC. (c–e) The expression level of WDR62 in different subtypes of BRCA in UALCAN, TISIDB, and TCGA portal databases.

Given the heterogeneity of tumor, single TCGA dataset could not reflect accurately the real expression level of genes. We firstly analyzed the expression level of WDR62 in 4 different databases—GEPIA2, TIMER, UALCAN, and StarBase. We discovered that WDR62 was overexpressed in over 20 tumor types (Figure 1(a) and S-Figure 1). The prognostic value was analyzed in 8 different databases—GEPIA2, TIMER, UALCAN, StarBase, TISIDB, TCGA portal, Kaplan-Meier Plotter, and PrognoScan. We selected 6 candidate tumors—bladder urothelial carcinoma (BLCA), breast invasive carcinoma (BRCA), kidney renal clear cell carcinoma (KIRC), kidney renal papillary cell carcinoma (KIRP), liver hepatocellular carcinoma (LIHC), and lung adenocarcinoma (LUAD), since WDR62 was overexpressed in at least 3 different databases and also had potential prognostic value in at least 2 databases among these tumors (Figures 1(b) and 1(c)). Moreover, we observed a correlation between WDR62 expression and the pathological stages of tumors, contributing to the diagnosis of tumor stage (Figures 2(a) and 2(b)). Notably, among different subtypes of BRCA, the expression level of WDR62 in triple-negative breast cancer (TNBC) was higher than other types including Her2, LumA, and LumB type (Figures 2(c) – 2(e)), implying the possibility that WDR62 participates in the tumorigenesis of TNBC. Additionally, WDR62 expression level was associated with tumor stage, subclass, histologic subtype, patient's age, and race in BRCA (S-Figure 2). In summary, WDR62 could serve as a diagnostic biomarker in most of the tumors.

### 3.2. Survival Analysis Data

**Figure 3:**
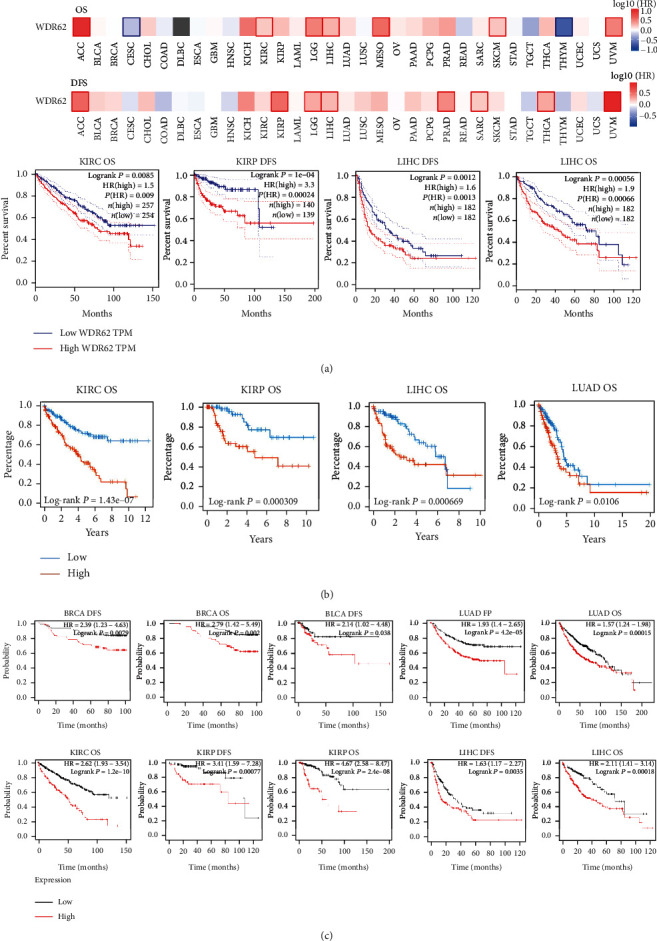
Correlation between WDR62 expression and survival prognosis of tumors. (a) The survival map of WDR62 was analyzed by GEPIA2. (b) Prognosis value—OS of KIRC, KIRP, LIHC, and LUAD in the TISIDB database. (c) The survival value of WDR62 using the Kaplan-Meier Plotter.

Subsequently, we investigated the prognosis value of WDR62 in different tumors. By GEPIA2 database, we performed the survival map of WDR62 across all tumors and analyzed the OS and DFS in detail in 6 candidate tumors (Figure 3(a)). Interestingly, high-expressed WDR62 indicated poor prognosis including OS and DFS of KIRC, KIRP, LIHC, and LUAD in databases based on TCGA datasets (Figure 3(b), S-Figure 3A–C). But there was no prognostic value in BLCA and BRCA even with higher WDR62 expression in these databases. However, it is notable that high-expressed WDR62 in basal-type BRCA implied poor OS in the TIMER database, suggesting different roles of WDR62 in BRCA subtypes (S-Figure 3B). We continued to analyze the survival data of WDR62 using Kaplan-Meier Plotter and PrognoScan databases based on GEO datasets. Unexpectedly, we identified a correlation between WDR62 expression and poor prognosis in BLCA and BRCA (Figure 3(c) and S-Figure 3D). Consequently, the above data indicated that WDR62 could serve as a prognostic biomarker in specific tumor types.

### 3.3. Driver Gene and Promoter Methylation Analysis Data

**Figure 4:**
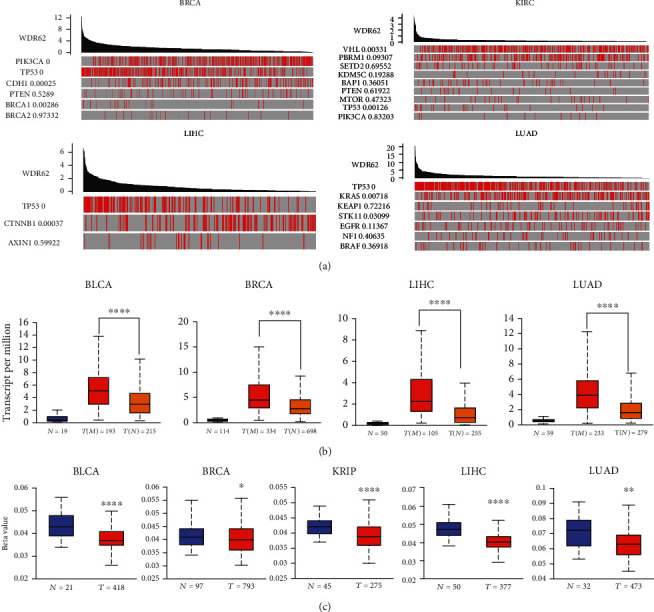
Correlation between WDR62 expression and driver gene and promoter methylation. (a) The value adjacent to highly mutated gene is permutation test *P* value of gene expression between driver mutated (red) and not mutated (gray) samples. (b) Correlation between WDR62 expression and mutated TP53 in the UALCAN database. (c) Correlation between WDR62 expression and promoter DNA methylation in the UALCAN database.

Now that WDR62 is overexpressed in most of the tumors, we are curious about the reasons. By TCGA portal database, we compared the correlation between WDR62 expression and important driver genes in BRCA, KIRC, LIHC, and LUAD, including PIK3CA, TP53, PTEN, BRCA1, MTOR, CTNNB1, KRAS, EGFR, and BRAF. As shown in Figure 4(a), TP53 showed the highest correlation in all these 4 tumors, providing a cause for WDR62 overexpression. And we confirmed that WDR62 expression was significantly associated with mutated TP53 in the UALCAN database, further validating the result (Figure 4(b)). Meanwhile, we observed an obvious negative correlation between WDR62 expression and its promoter DNA methylation (Figure 4(c)), suggesting another possibility.

### 3.4. Enrichment Analysis of WDR62-Related Genes

**Figure 5:**
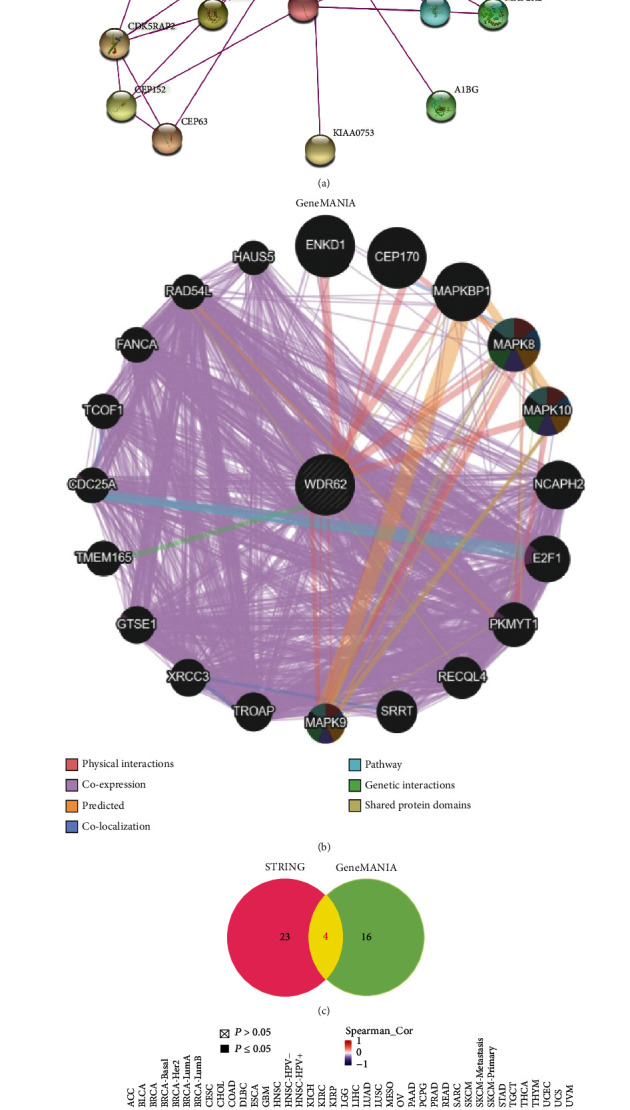
WDR62-related gene enrichment analysis. (a) The available experimentally determined WDR62-binding proteins using the STRING tool. (b) Using the GeneMANIA approach, top 20 WDR62-correlated genes were analyzed. (c) An intersection analysis of the WDR62-binding and correlated genes was conducted. (d) The corresponding heat map data in the detailed cancer types is displayed. (e) GEPIA2 was used to analyze the correlation between WDR62 and selected targeting genes, including CEP170, MAPK8, MAPK9, and MAPK10.

To further explore the molecular mechanism of WDR62 in carcinogenesis, we screened out WDR62-binding proteins by STRING online tool. We acquired a total of 27 genes supported by experimental evidence (Figure 5(a)). Subsequently, we investigated the interaction network for WDR62 by the GeneMANIA database.  Figure 5(b) shows the top 20 genes and their relationship with WDR62. We compared the two sets of genes and discovered 4 common members—CEP170, MAPK8, MAPK9, and MAPK10 (Figure 5(c)). Then, we used the GEPIA2 database to obtain the correlation heat map data in all tumor types (Figure 5(d)). As shown in Figure 5(e), WDR62 expression was positively correlated with that of CEP170, MAPK8, and MAPK9 in the majority of tumors. In contrast, WDR62 expression was negatively correlated with that of MAPK10 in the majority of tumors. Finally, we combined the two groups of genes and performed GO enrichment and KEGG pathway analysis via DAVID online tool (S-Figure 4A–D). Results demonstrated that WDR62 relative genes may be mainly involved in the MAPK signaling pathway (S-Figure 4E).

### 3.5. Immune Infiltration Analysis Data

**Figure 6:**
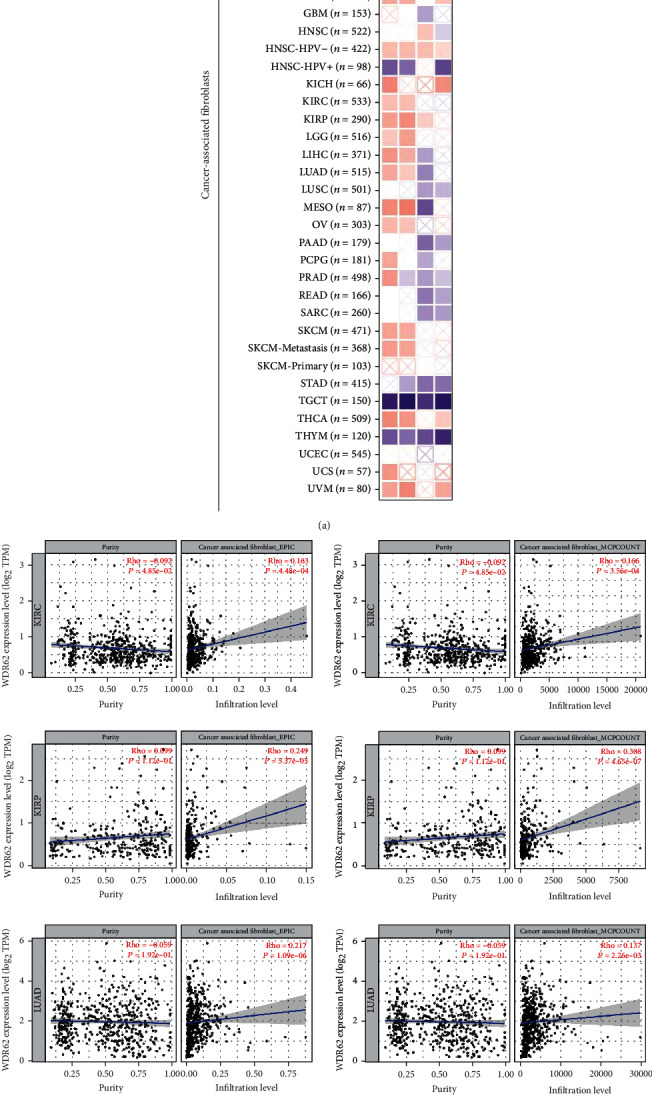
Correlation analysis between WDR62 expression and immune infiltration of CAF. (a) The heat map about correlation between WDR62 expression and cancer-associated fibroblasts (CAF). (b) The correlation between WDR62 expression and cancer-associated fibroblasts (CAF) in BLCA, KIRC, KIRP, LIHC, and LUAD.

**Figure 7:**
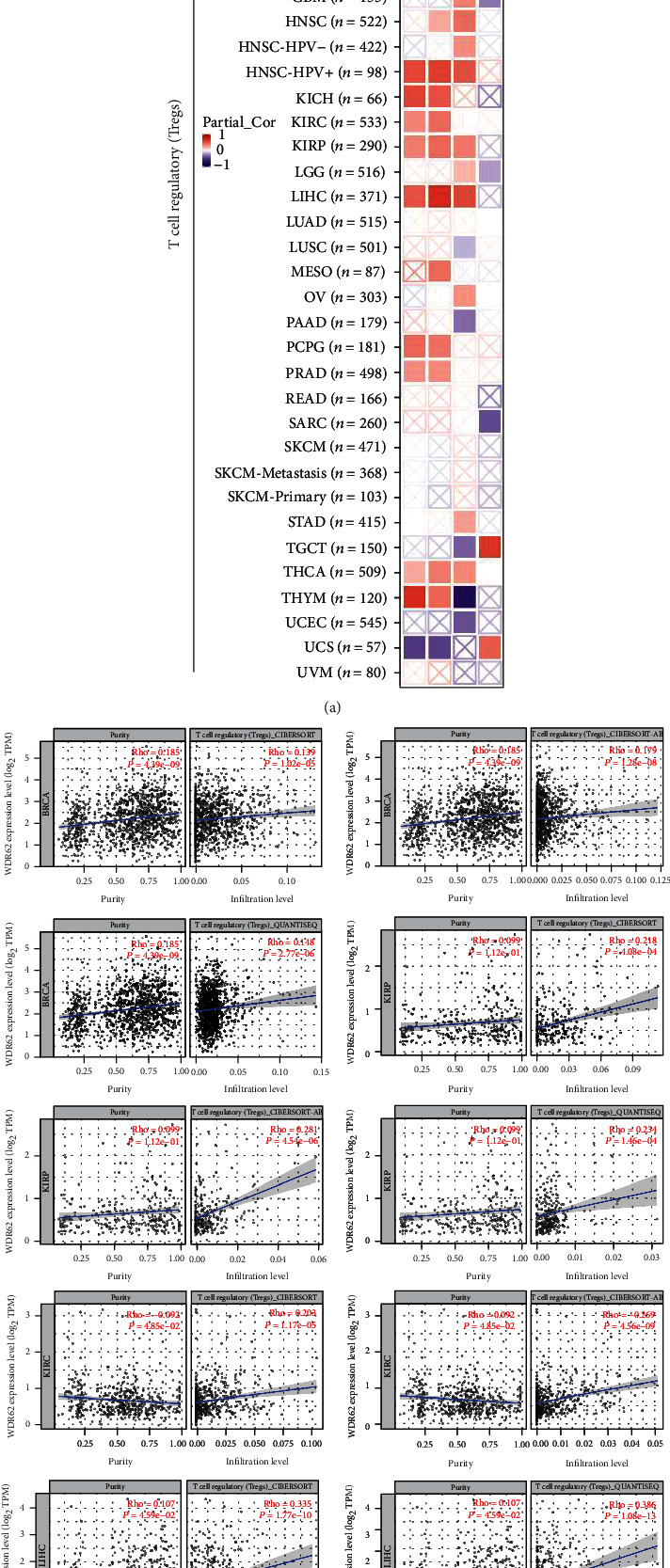
Correlation analysis between WDR62 expression and immune infiltration of Treg cells. (a) The heat map about correlation between WDR62 expression and Treg cells. (b) The correlation between WDR62 expression and Treg cells in BRCA, KIRC, KIRP, and LIHC.

**Figure 8:**
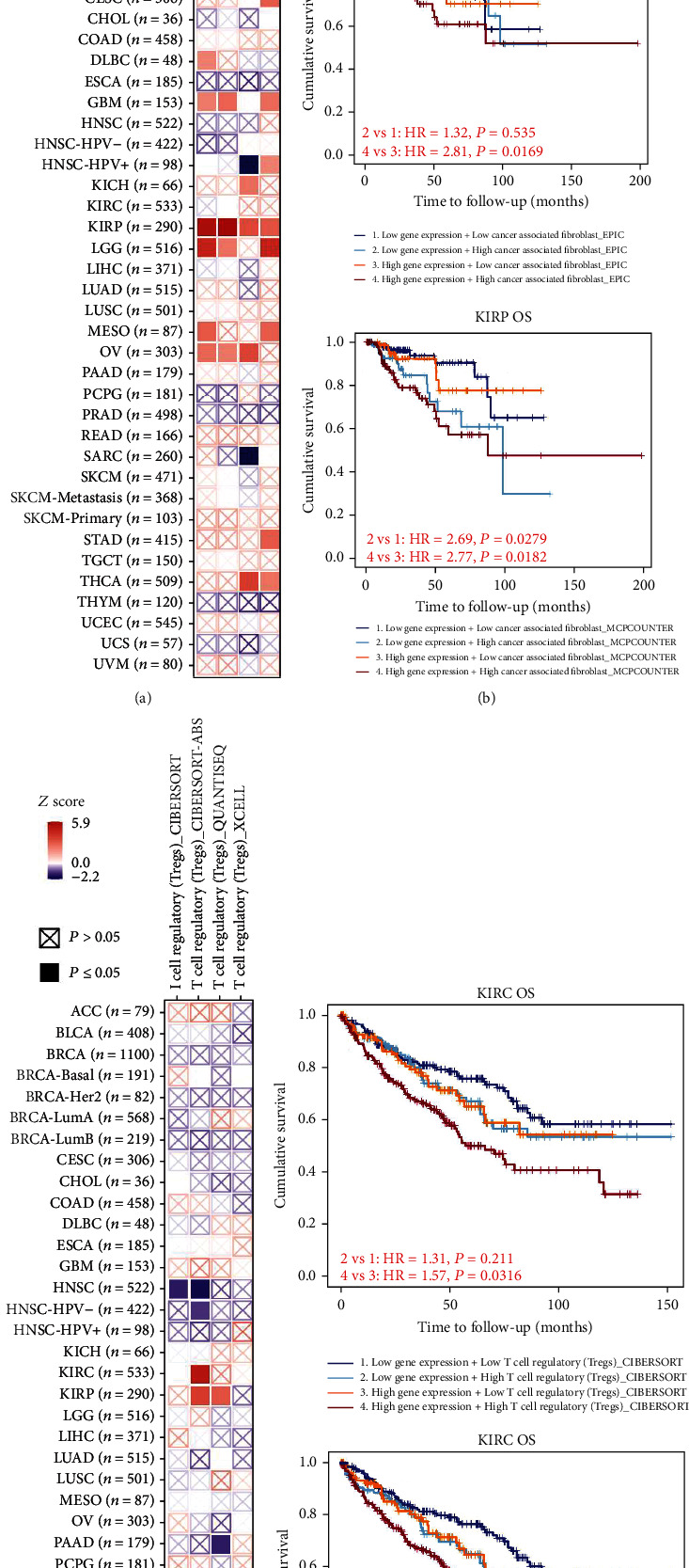
Survival prognosis between WDR62 expression and CAF or Treg cell infiltration. (a) The heat map about OS between WDR62 expression and cancer-associated fibroblasts (CAF). (b) OS between WDR62 expression and cancer-associated fibroblasts (CAF) in KIRP. (c) The heat map about OS between WDR62 expression and T cell regulatory (Treg) cells. (d) OS between WDR62 expression and T cell regulatory (Treg) cells in KIRC.

Other than the MAPK pathway, WDR62 was also closely correlated with the toll-like receptor signaling pathway, which is involved in immune reaction. Therefore, we are keen to explore the association between WDR62 expression and immune features. Firstly, we analyzed a series of immune cells via the TIMER database in 6 candidate tumors. The results showed that WDR62 expression was closely correlated with the infiltration level of several immune cells, including B cell, CD4+ T cell, CD8+ T cell, macrophage, neutrophil, and dendritic cell (S-Figure 5). More importantly, WDR62 expression was associated with the abundance of CAF and Treg cells by using TIMER2 platform analysis (Figures 6 and 7). Besides, we observed that the prognosis of patients with high WDR62 expression plus high CAF or Treg is the worst in KIRP and KIRC, respectively (Figure 8). GEO datasets also indicated that high WDR62 expression predicted worse prognosis with high Treg cell infiltration or low immune cell infiltration such as CD4+ T cell, CD8+ T cell, macrophage, and NK cell (S-Figure 6).

### 3.6. Association Analysis with Immunomodulators

**Figure 9:**
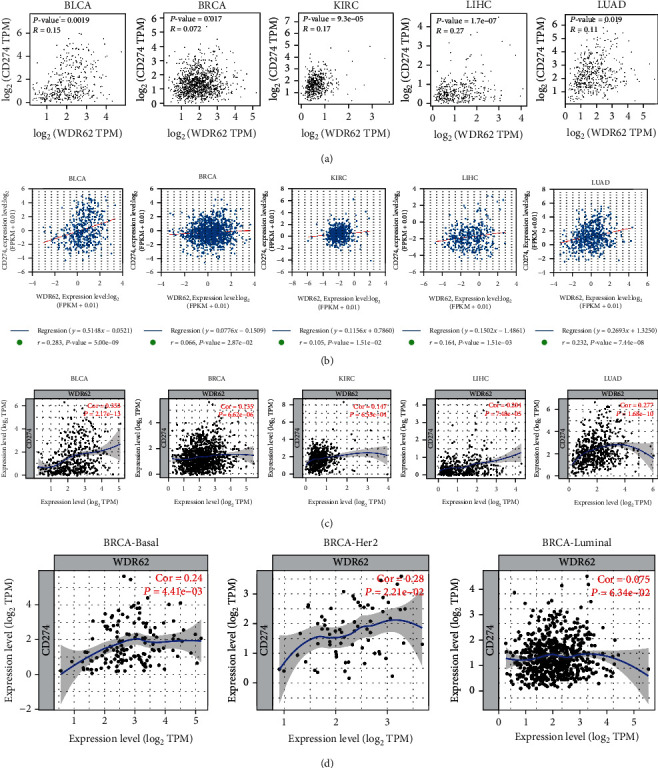
Correlation analysis between WDR62 expression and PD-L1 expression. (a–c) The correlation between WDR62 expression and PD-L1 (CD274) expression was analyzed in the GEPIA2, StarBase, and TIMER databases. (d) The correlation between WDR62 expression and PD-L1 (CD274) expression was analyzed in different subtypes of BRCA.

**Figure 10:**
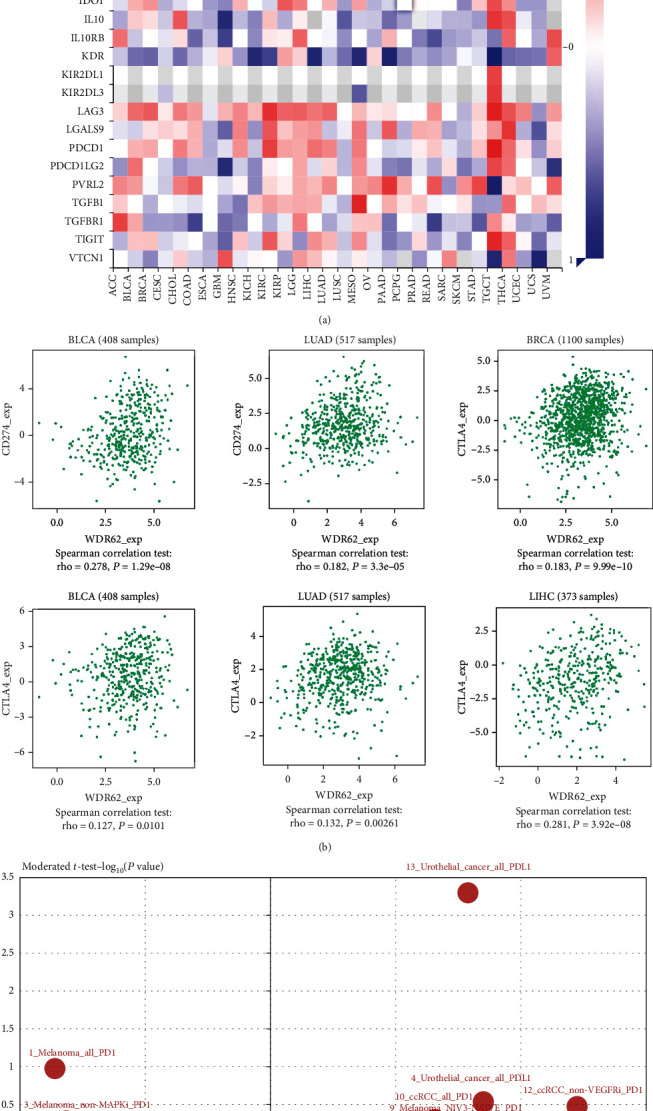
Association analysis with immunomodulators. (a) The relationship between WDR62 and immunoinhibitors was analyzed by Spearman's correlation analysis via the TISIDB database. (b) The correlation between WDR62 expression and PD-L1 or CTLA4 expression in BLCA, BRCA, LUAD, and LIHC. (c) WDR62 expression difference between responders and nonresponders receiving PD1/PD-L1 inhibitors.

With the popularity of immunotherapy, in particular PD1/PD-L1 inhibitors, we wondered whether WDR62 was relevant with immunomodulators. By coincidence, in 3 databases, the expression of WDR62 was positively correlated with PD-L1 (CD274) expression in 5 candidate tumors except KIRP (Figures 9(a) – 9(c)). Notably, WDR62 expression in triple-negative breast cancer (TNBC) was more correlated with PD-L1 expression than Her2 and luminal subtype (Figure 9(d)), in accordance with the above data about WDR62's role in TNBC. To further investigate the relationship between WDR62 and immunomodulators, we performed Spearman's correlation analysis via the TISIDB database. We found that WDR62 expression was mostly positively correlated with the abundance of immunoinhibitors such as PD-L1 and CTLA4 (Figures 10(a) and 10(b)). Finally, we discovered that WDR62 had significant difference of expression between responders and nonresponders receiving atezolizumab (anti-PD-L1) in urothelial cancer (Figure 10(c)). Collectively, these results demonstrated that WDR62 may indeed be involved in tumor immune regulation.

## 4. Discussion

Despite the great advances in diagnosis and treatment of tumors, it is still threatening human survival. Hence, there is an urgent need to identify new tumor biomarkers beneficial for early diagnosis of tumor and the prediction of cancer therapeutic effect. It has been reported that WDR62 plays crucial roles in many cellular processes such as spindle maintenance, cell cycle progression, and cell proliferation [21–23]. Recently, accumulating evidence suggests that WDR62 is a new identified tumor biomarker in few tumors such as lung cancer and bladder cancer [24, 25]. Shinmura et al. suggested WDR62 was overexpressed in LUAD and was associated with a poor prognosis. In order to figure out the oncogenic role of WDR62, we performed a pan-cancer analysis from the view of overall tumors based on bioinformatic data.

In this study, we identified that WDR62 could serve as a diagnostic biomarker in most of the tumors since WDR62 was overexpressed simultaneously in at least 3 different databases. In breast cancer, with the highest morbidity now, we further analyzed the datasets and discovered a correlation between WDR62 expression and clinical features. More importantly, we observed that WDR62 expression in triple-negative breast cancer (TNBC) was higher than that in other subtypes, suggesting that a distinct mechanism may exist for WDR62 in BRCA subtypes. Subsequently, our research indicated that WDR62 could also serve as a prognostic biomarker mainly in 6 tumor types. Among them, KIRC, KIRP, and LIHC showed the best prognostic value due to its significance in most databases. Though BLCA and BRCA did not have prognostic value via TCGA-based databases, we found a survival difference in GEO-based databases, suggesting that there was a discrepancy about prognostic value in different databases based on TCGA or GEO datasets. Extraordinarily, our study demonstrated that high-expressed WDR62 implied poor survival in TNBC, further highlighting its potential as a biomarker in TNBC.

The research on the molecular mechanism and functional enrichment analysis of WDR62 in tumors is seldom. For instance, Sugita et al.'s study reported that microRNA-223 was responsible for the abnormal expression of WDR62 in bladder cancer [25]. Therefore, we first applied TCGA datasets to explore the molecular mechanism for WDR62 high expression. Our findings demonstrated high-expressed WDR62 was subject to multiple driver genes in particular TP53 which showed the highest correlation in all candidate tumors. It is of interest to further explore the mechanism between WDR62 and TP53 in future study. Besides, we discovered a correlation between WDR62 expression and its promoter DNA methylation, providing another cause for WDR62 high expression. Subsequently, we integrated the information about WDR62-binding proteins and WDR62-relevant genes for enrichment analysis and identified the potential effect on the MAPK and toll-like receptor signaling pathway, which was consistent with previous findings that JNK, one MAPK cascade, was activated by WDR62 in tumor cells [26]. More importantly, we are the first study to present evidence about the correlation between WDR62 expression and immune cell infiltration in 6 candidate tumors. Extraordinarily, a significant positive correlation was suggested between WDR62 expression and the abundance of CAF and Treg cells. And the patients with high WDR62 expression plus high CAF or Treg cell infiltration have the worst overall survival. The prominent components of tumor microenvironment are immune cells which were related with tumorigenesis, progression, and metastasis [27, 28]. Accordingly, we considered that WDR62 may play vital roles in tumorigenesis by regulating the tumor microenvironment (TM). By coincidence, we found a significant expression of exosomal WDR62 in colorectal cancer, hepatocellular carcinoma, and pancreatic adenocarcinoma by the exoRBase database (S-Figure 4F). Given that exosomes exhibit their property in intercellular communication between tumor cells and tumor microenvironment [29], we inferred that the regulatory effect on CAF or Treg cells may be by means of exosomal WDR62.

Immunotherapy in particular PD1/PD-L1 inhibitors has aroused great interest in recent years. Detecting PD1/PD-L1 expression levels by immunohistochemistry was treated as standard assay for applying PD1/PD-L1 inhibitors [30]. However, there are significant differences in responsiveness and efficacy of candidate patients even with the same tumor subtype. Tumor mutation burden (TMB), another biomarker to predict immunotherapy response, is also unsatisfactory which is attributed to its high expense and being time-consuming [31]. Therefore, new predictive biomarkers for immunotherapy are indispensable for selecting suitable patients and studying innate or acquired resistance. As the most malignant subtype of breast cancer, triple-negative breast cancer (TNBC) received only mild responses in monotherapy with PD1/PD-L1 inhibitors [32]. However, given its higher infiltration level of immune cells, TNBC should have benefited from immunotherapies. Our study presented evidence about the correlation between WDR62 expression and PD-L1 expression in 5 candidate tumors. More importantly, we revealed that WDR62 expression had a marked correlation with PD-L1 expression in TNBC. Therefore, we wonder whether it is suitable to predict the effect of PD1/PD-L1 inhibitors by the detection of WDR62 expression. Based on the TISIDB database, we observed that WDR62 had a significant difference of expression between responders and nonresponders receiving atezolizumab (anti-PD-L1) in urothelial cancer. Collectively, WDR62 could reflect some immune status and indeed could be a predictive biomarker for PD1/PD-L1 inhibitors in some tumor type.

However, our study still has several limits. First, online databases have limitations because different databases produce distinct results due to various sample types and sizes. Moreover, our study showed bioinformatic analysis findings which need further experiments to validate. Additionally, there are many questions to be solved. For example, the underlying molecular mechanism between WDR62 expression and TP53 mutation needs to be explored. How to influence the function of CAF or Treg cells by exosomal WDR62? What about the effect of WDR62 expression on the prediction for immunotherapy in other tumor types? In brief, our first pan-cancer analysis of WDR62 suggested that WDR62 was overexpressed in multiple tumors and closely correlated with poor prognosis and immune regulation, contributing to understanding the oncogenic role of WDR62 across human tumors.

## 5. Conclusions

Our study revealed that WDR62 could serve as a diagnostic and prognostic biomarker for several cancers. Importantly, WDR62 was closely associated with various immune cell infiltration, and to a certain extent, it can predict the effect of immunotherapy in particular PD1/PD-L1 inhibitors. Our pan-cancer study provided useful information on the oncogenic role of WDR62, contributing to further exploring the underlying mechanisms.

## Data Availability

All databases including GEPIA2, TIMER, UALCAN, StarBase, TISIDB, TCGA portal, Kaplan-Meier Plotter, and PrognoScan are freely available as public resources.

## Abbreviations

BLCA: Bladder urothelial carcinoma
BP: Biological process
BRCA: Breast invasive carcinoma
CAF: Cancer-associated fibroblasts
CC: Cellular component
DAVID: Database for Annotation, Visualization, and Integrated Discovery
DFS: Disease-free survival
DSS: Disease-specific survival
FP: First progression survival
GEO: Gene Expression Omnibus
GEPIA: Gene Expression Profiling Interactive Analysis
GO: Gene Ontology
HR: Hazard ratio
IARC: International Agency for Research on Cancer
JNK: c-Jun N-terminal kinase
KEGG: Kyoto Encyclopedia of Genes and Genomes
KIRC: Kidney renal clear cell carcinoma
KIRP: Kidney renal papillary cell carcinoma
LIHC: Liver hepatocellular carcinoma
LUAD: Lung adenocarcinoma
MAPK: Mitogen-activated protein kinases
MF: Molecular function
NK: Natural killer T cells
OS: Overall survival
PD1: Programmed cell death 1
PD-L1: Programmed cell death 1 ligand 1
PPI: Protein-protein interaction
PPS: Relapse survival
TCGA: The Cancer Genome Atlas
TIMER: Tumor Immune Evaluation Resource
TM: Tumor microenvironment
TMB: Tumor mutation burden
TNBC: Triple-negative breast cancer
Treg: T cell regulatory
WDR62: WD repeat domain 62.

## Supplementary Materials

Supplementary 1 S-Figure 1: expression level of WDR62 in different tumors. The expression level of WDR62 in different tumors was analyzed through the GEPIA2 database.

Supplementary 2 S-Figure 2: the correlation between WDR62 expression level and clinical factors in BRCA. WDR62 expression level was analyzed in BRCA tumor stage (A), subclass (B), histologic subtype (C), patient's age (D), patient's race (E), and nodal metastasis status (F) by the UALCAN database.

Supplementary 3 S-Figure 3: correlation between WDR62 expression and survival prognosis of tumors. (A) Prognosis value—OS of KIRC, KIRP, LIHC, and LUAD in the UALCAN database. (B) Prognosis value—OS of KIRC, LIHC, and BRCA-basal in the TIMER database. (C) Prognosis value—OS of KIRC, KIRP, and LIHC in TCGA portal database. (D) The survival value of WDR62 using the PrognoScan database.

Supplementary 4 S-Figure 4: GO and KEGG analysis. (A–D) Based on the WDR62-binding and interacted genes, GO analysis—biological process (BP), molecular function (MF), and cellular component (CC)—and KEGG pathway analysis were performed. (E) The molecular function data in GeneMANIA is shown. (F) WDR62 gene expression profiles in exosome by the exoRBase database.

Supplementary 5 S-Figure 5: correlation analysis between WDR62 expression and immune cell infiltration in 6 candidate tumors—BLCA, BRCA, KIRC, KIRP, LIHC, and LUAD.

Supplementary 6 S-Figure 6: survival prognosis between WDR62 expression and immune cell infiltration. Survival data between WDR62 expression and immune cell infiltration, including CD4+ T cell, CD8+ T cell, macrophage, natural killer (NK) T cells, and regulatory T cells (Treg), in BLCA, BRCA, KIRC, LIHC, and LUAD using the Kaplan-Meier Plotter.
