# Supplementary figures and images for: Systematic Analysis of the Oncogenic Role of WDR62 in Human Tumors
Source: Dis Markers. 2021 Jul 1;2021:9940274. doi: 10.1155/2021/9940274 (PMC8272457; doi:10.1155/2021/9940274)

## Slide 1
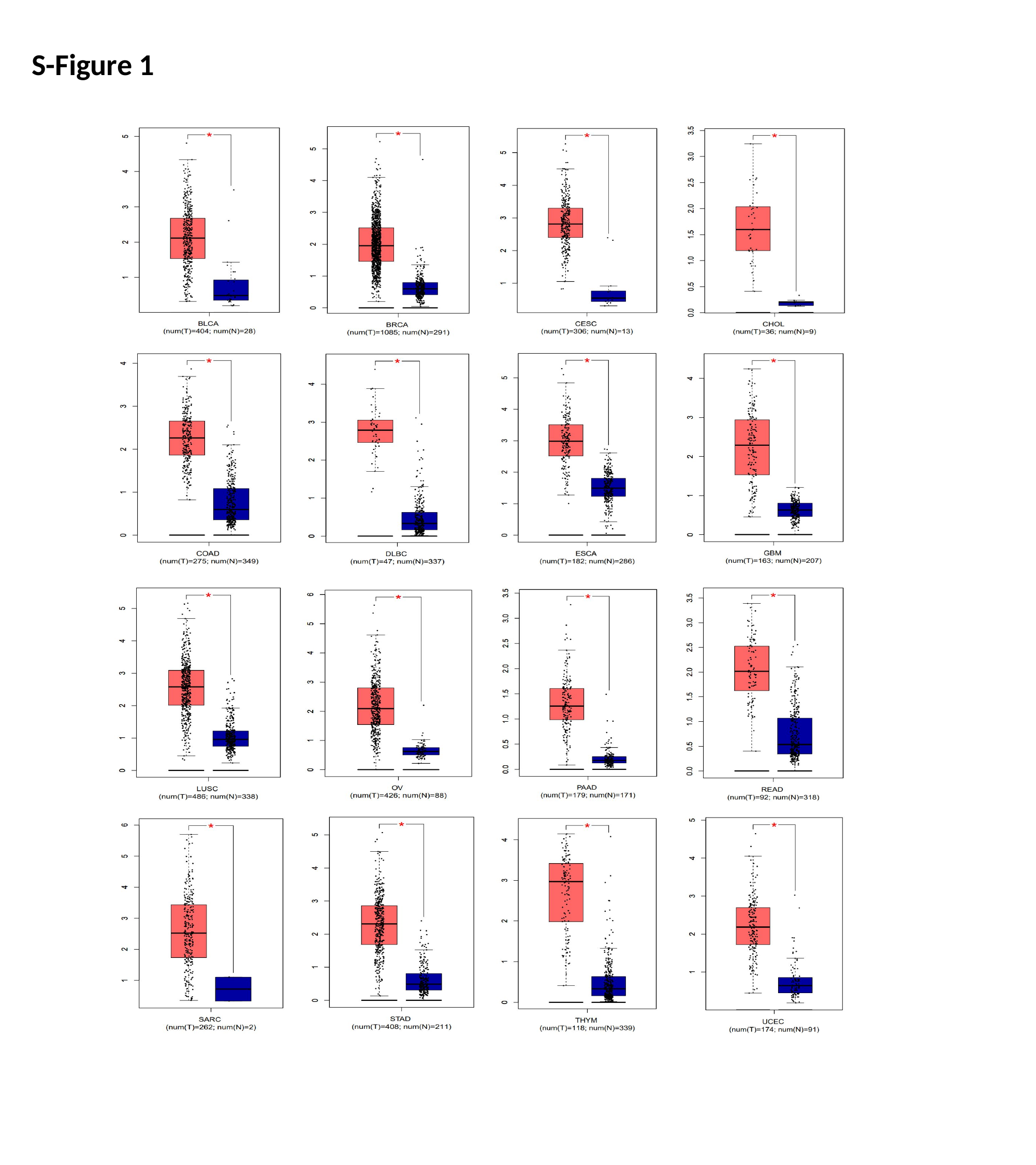

S-Figure 1

Supplement: Supplementary 1 — S-Figure 1: expression level of WDR62 in different tumors. The expression level of WDR62 in different tumors was analyzed through the GEPIA2 database. [file 9940274.f1.pptx]

## Slide 1
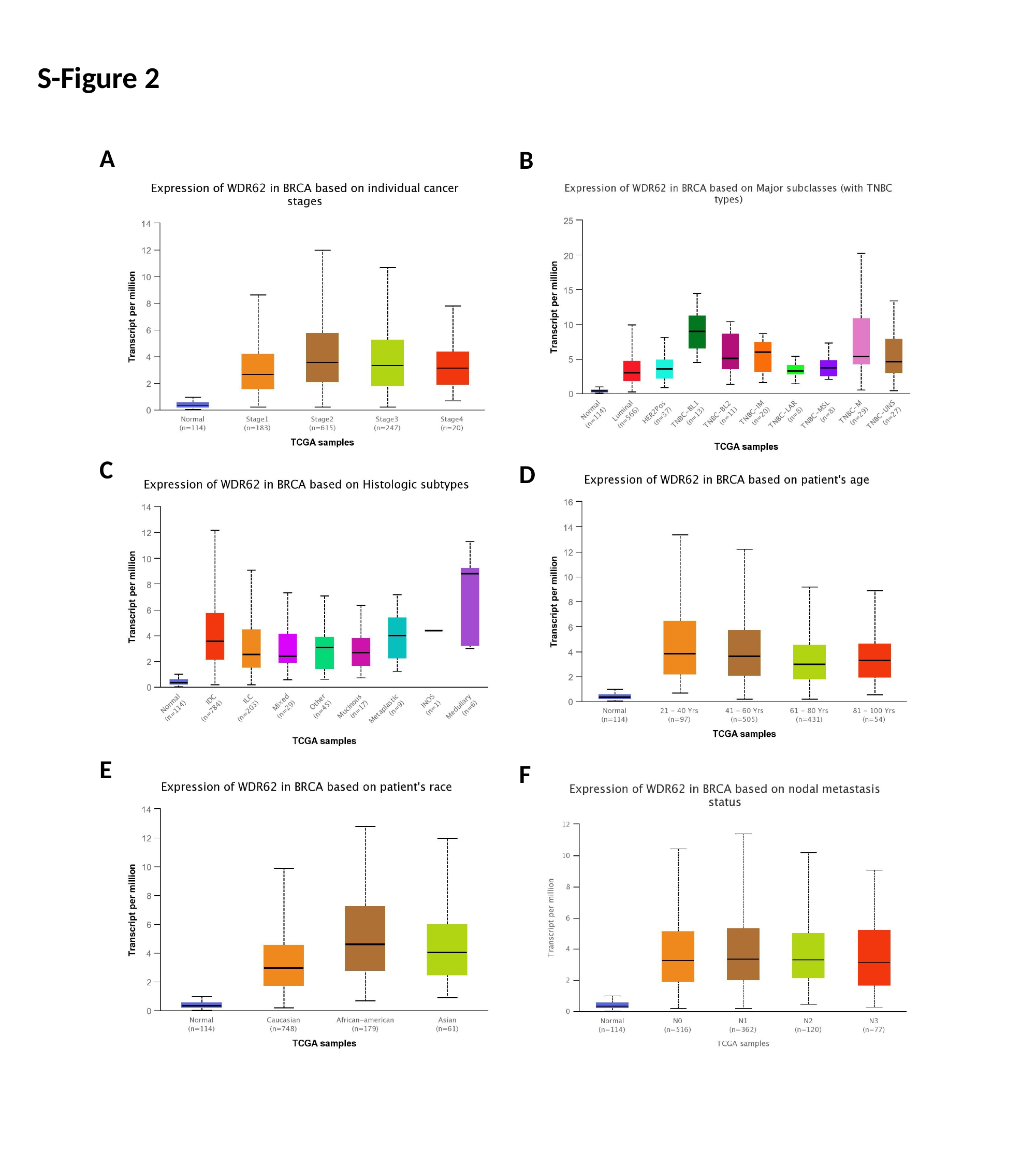

S-Figure 2
A
B
C
D
E
F

Supplement: Supplementary 2 — S-Figure 2: the correlation between WDR62 expression level and clinical factors in BRCA. WDR62 expression level was analyzed in BRCA tumor stage (A), subclass (B), histologic subtype (C), patient's age (D), patient's race (E), and nodal metastasis status (F) by the UALCAN database. [file 9940274.f2.pptx]

## Slide 1
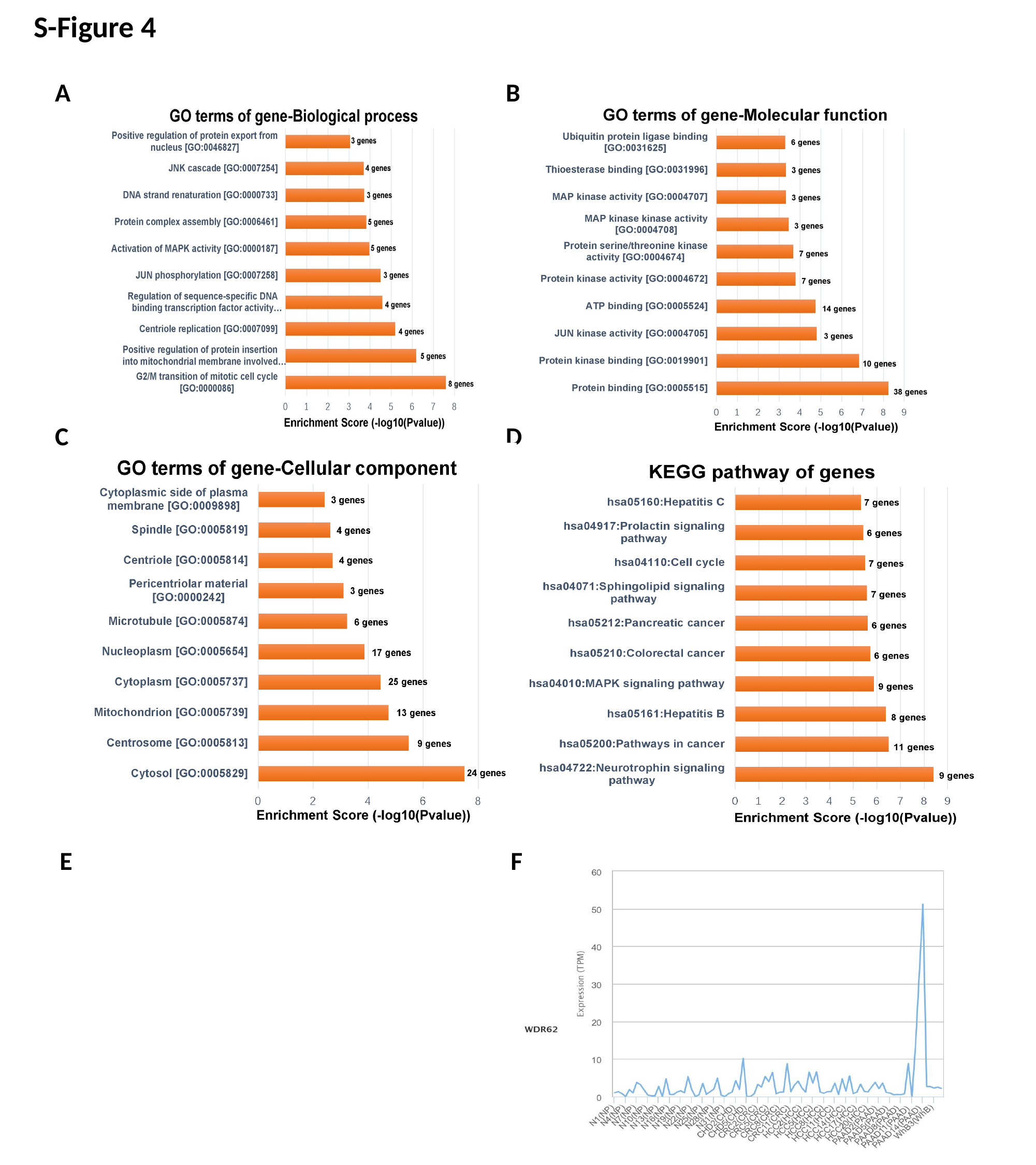

S-Figure 4
A
B
C
D
E
F

Supplement: Supplementary 4 — S-Figure 4: GO and KEGG analysis. (A–D) Based on the WDR62-binding and interacted genes, GO analysis—biological process (BP), molecular function (MF), and cellular component (CC)—and KEGG pathway analysis were performed. (E) The molecular function data in GeneMANIA is shown. (F) WDR62 gene expression profiles in exosome by the exoRBase database. [file 9940274.f4.pptx]

## Slide 1
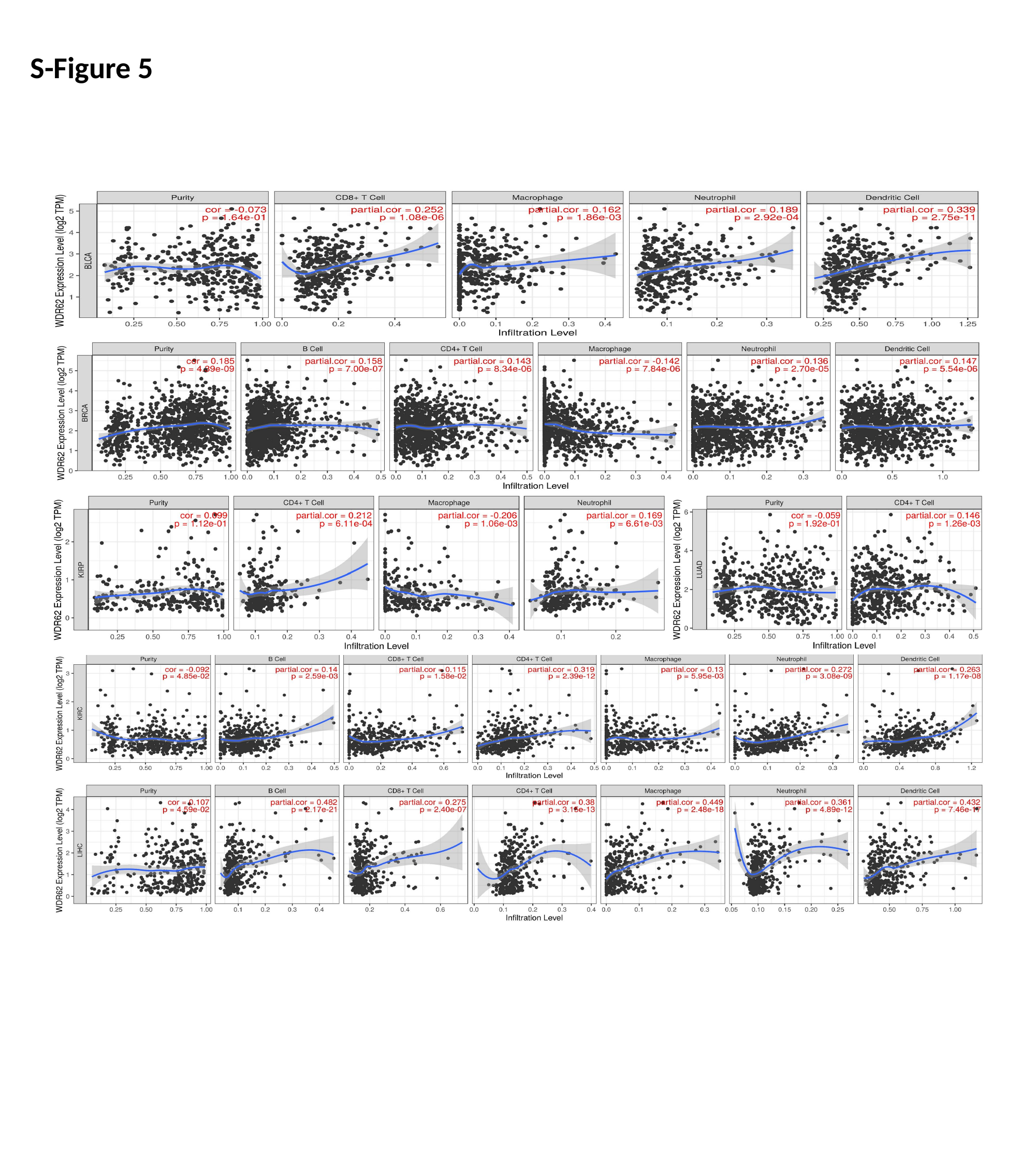

S-Figure 5

Supplement: Supplementary 5 — S-Figure 5: correlation analysis between WDR62 expression and immune cell infiltration in 6 candidate tumors—BLCA, BRCA, KIRC, KIRP, LIHC, and LUAD. [file 9940274.f5.pptx]
