# Supplementary material for: Systematic Analysis of the Oncogenic Role of WDR62 in Human Tumors
Source: Dis Markers. 2021 Jul 1;2021:9940274. doi: 10.1155/2021/9940274 (PMC8272457; doi:10.1155/2021/9940274)
Supplement: Supplementary 3 — S-Figure 3: correlation between WDR62 expression and survival prognosis of tumors. (A) Prognosis value—OS of KIRC, KIRP, LIHC, and LUAD in the UALCAN database. (B) Prognosis value—OS of KIRC, LIHC, and BRCA-basal in the TIMER database. (C) Prognosis value—OS of KIRC, KIRP, and LIHC in TCGA portal database. (D) The survival value of WDR62 using the PrognoScan database. [file 9940274.f3.pptx]

## Slide 1
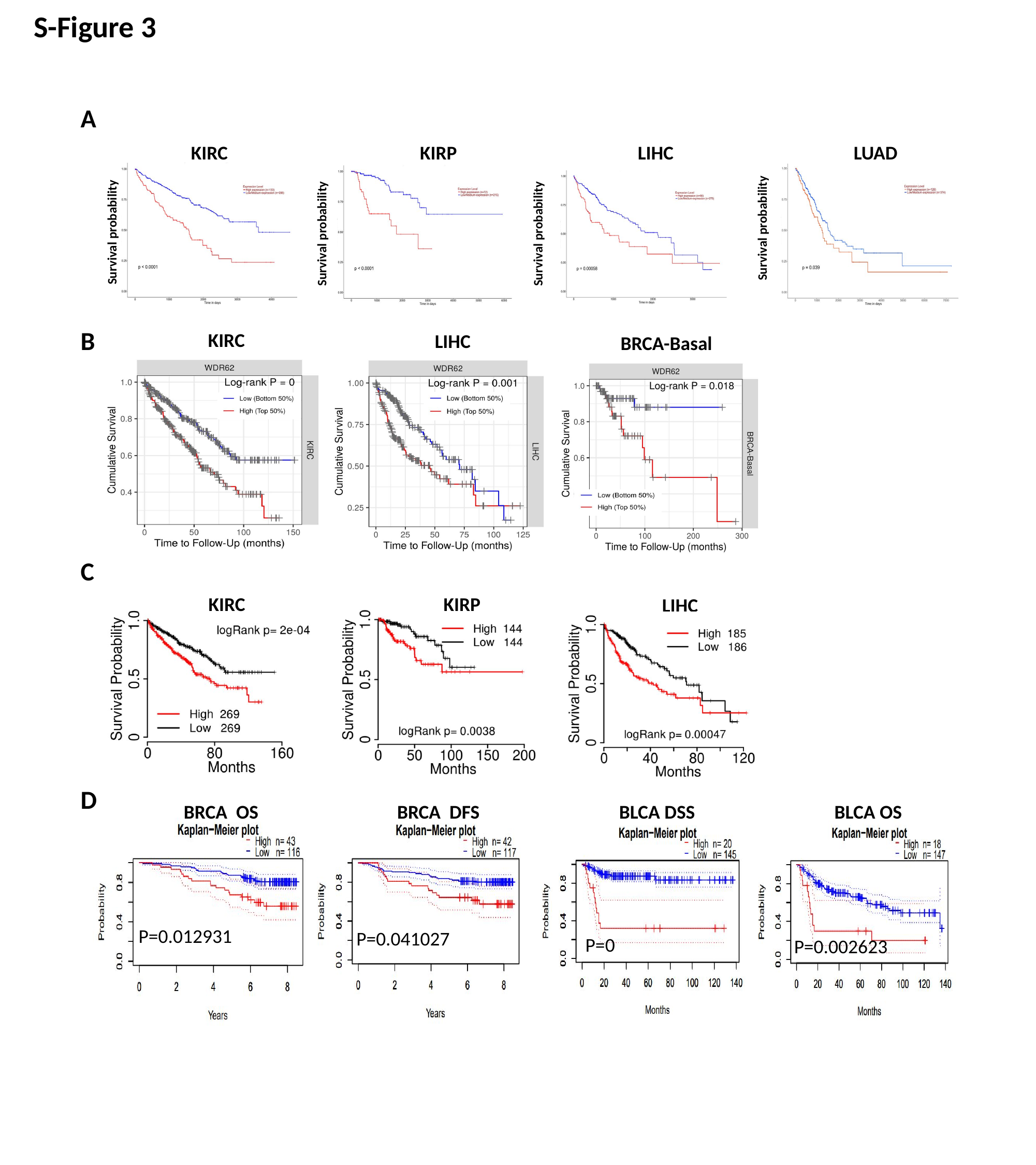

S-Figure 3
A
KIRC
KIRP
LIHC
LUAD
Survival probability
Survival probability
Survival probability
Survival probability
B
KIRC
LIHC
BRCA-Basal
C
KIRC
KIRP
LIHC
D
BRCA OS
BRCA DFS
BLCA DSS
BLCA OS
P=0.012931
P=0.041027
P=0
P=0.002623
