# Supplementary material for: Systematic Analysis of the Oncogenic Role of WDR62 in Human Tumors
Source: Dis Markers. 2021 Jul 1;2021:9940274. doi: 10.1155/2021/9940274 (PMC8272457; doi:10.1155/2021/9940274)
Supplement: Supplementary 6 — S-Figure 6: survival prognosis between WDR62 expression and immune cell infiltration. Survival data between WDR62 expression and immune cell infiltration, including CD4+ T cell, CD8+ T cell, macrophage, natural killer (NK) T cells, and regulatory T cells (Treg), in BLCA, BRCA, KIRC, LIHC, and LUAD using the Kaplan-Meier Plotter. [file 9940274.f6.pptx]

## Slide 1
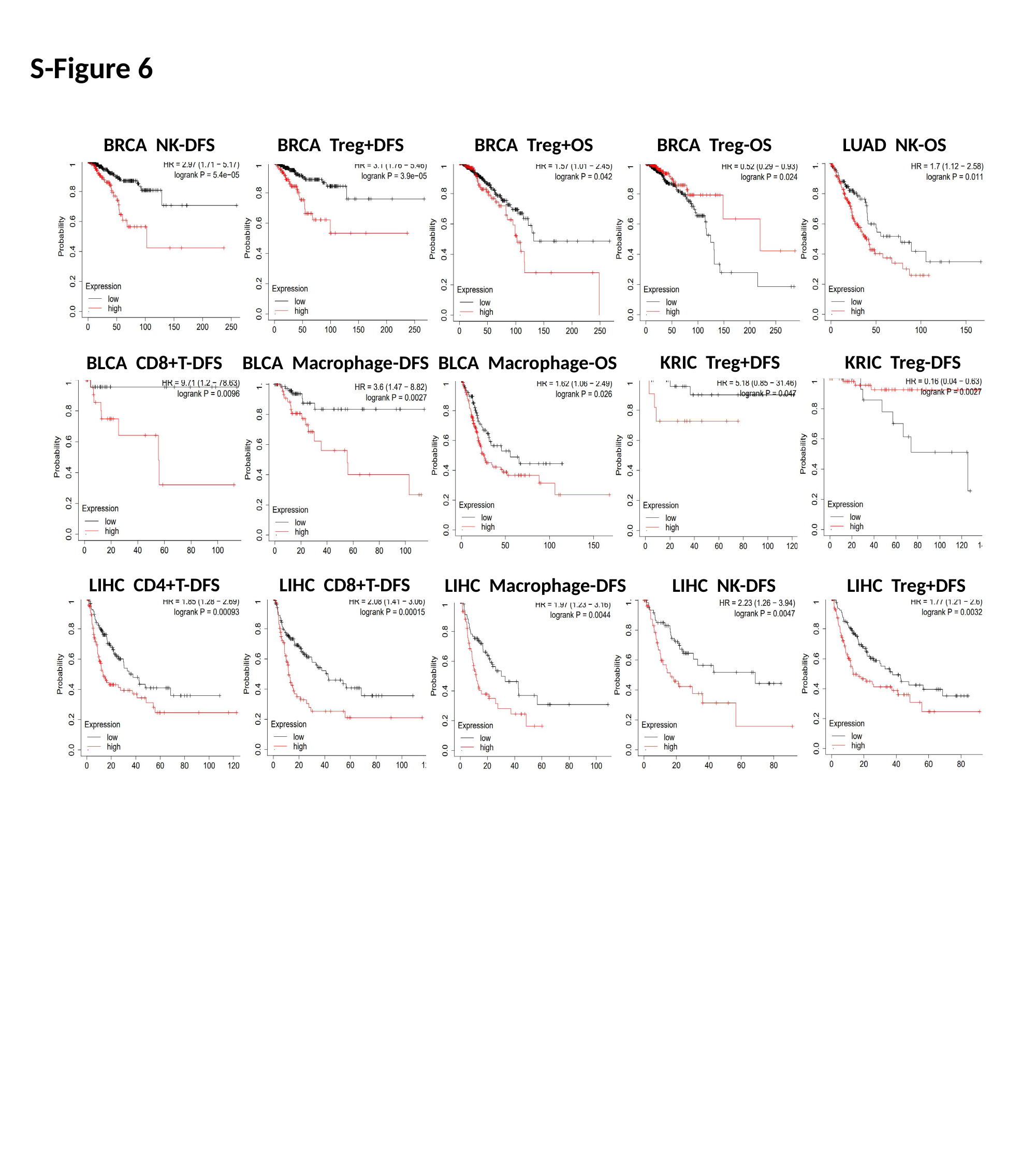

S-Figure 6
LUAD NK-OS
BRCA NK-DFS
BRCA Treg+DFS
BRCA Treg+OS
BRCA Treg-OS
KRIC Treg+DFS
KRIC Treg-DFS
BLCA CD8+T-DFS
BLCA Macrophage-DFS
BLCA Macrophage-OS
LIHC CD4+T-DFS
LIHC CD8+T-DFS
LIHC Macrophage-DFS
LIHC NK-DFS
LIHC Treg+DFS
